# Investigating Mass Transfer and Reaction Engineering Characteristics in a Membrane Biofilm Using *Cupriavidus necator* H16

**DOI:** 10.3390/membranes13120908

**Published:** 2023-12-14

**Authors:** Burcu Akkoyunlu, Sorcha Daly, Federico Cerrone, Eoin Casey

**Affiliations:** 1 School of Chemical and Bioprocess Engineering, University College Dublin, D04 V1W8 Dublin, Ireland; burcu.akkoyunlu@ucdconnect.ie (B.A.); s.b.daly@greenwich.ac.uk (S.D.); 2 BiOrbic Bioeconomy SFI Research Centre, University College Dublin, D04 V1W8 Dublin, Ireland; federico.cerrone@dcu.ie; 3 School of Engineering, Faculty of Engineering and Science, University of Greenwich, Medway Campus, Chatham ME4 4AG, UK; 4 UCD Earth Institute, School of Biomolecular and Biomedical Sciences, University College Dublin, D04 V1W8 Dublin, Ireland; 5 School of Biotechnology, Dublin City University, Glasnevin Campus, D09 N920 Dublin, Ireland

**Keywords:** membrane biofilm reactor, Cupriavidus necator, oxygen transfer, mass transfer model, biofilm

## Abstract

Membrane biofilm reactors are a growing trend in wastewater treatment whereby gas-transfer membranes provide efficient bubbleless aeration. Recently, there has been a growing interest in using these bioreactors for industrial biotechnology using microorganisms that can metabolise gaseous substrates. Since gas fermentation is limited by the low solubilities of gaseous substrates in liquid media, it is critical to characterise mass transfer rates of gaseous substrates to enable the design of membrane biofilm reactors. The objective of this study is to measure and analyse mass transfer rates and reaction engineering characteristics for a single tube membrane biofilm reactor using *Cupriavidus necator* H16. At elevated Reynolds numbers, the dominant resistance for gas diffusion shifts from the liquid boundary layer to the membrane. The biofilm growth rate was observed to decrease after 260 μm at 96 h. After 144 h, some sloughing of the biofilm occurred. Oxygen uptake rate and substrate utilisation rate for the biofilm developed showed that the biofilm changes from a single-substrate limited regime to a dual-substrate-limited regime after 72 h which alters the localisation of the microbial activity within the biofilm. This study shows that this platform technology has potential applications for industrial biotechnology.

## 1. Introduction

**Figure 1:**
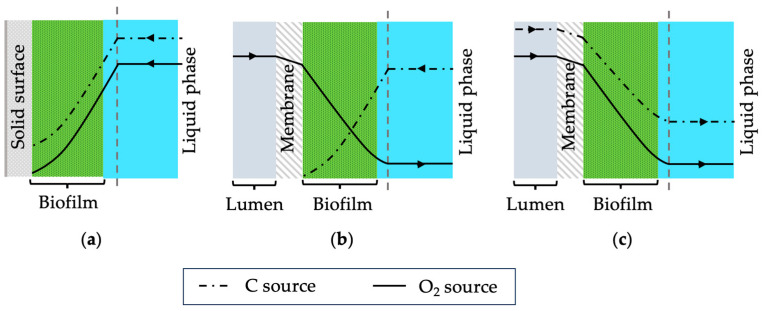
A schematic of different nutrient profiles for biofilms formed on (**a**) a solid surface, (**b**) a membrane used for aeration, and (**c**) a membrane used for supplying gaseous substrate.

The membrane biofilm reactor, sometimes called a membrane aerated biofilm reactor, is an emerging reactor system for wastewater treatment [1]. In such systems, biofilms form on gas-permeable membranes where gaseous substrates such as oxygen diffuse through the membrane into the biofilm. Conventionally, biofilms grow on static surfaces where nutrients are delivered from the biofilm-liquid interface (see Figure 1a). In membrane biofilm reactors, substrates are delivered from both sides, resulting in distinct nutrient profiles. Depending on the concentration of nutrients and oxygen, the location of the biocatalytic activity within the biofilms can be controlled [2]. This influences the overall performance of the membrane biofilm reactors. Recently, these bioreactor systems have been investigated to produce various chemicals such as volatile fatty acids and ethanol using mixed-culture systems [3]. However, there is limited information on using pure cultures in membrane biofilm reactors.

For industrial biotechnology applications, a defined microbial species is used within the membrane bioreactor [3]. Although high product purities can be obtained compared to mixed cultures, using pure culture faces challenges such as contamination and sensitivity to gas composition supplied through the membrane [4]. In the scientific literature, methane-oxidising bacteria (methanotrophs), nitrate-reducing bacteria and acetogenic bacteria have been studied extensively in membrane biofilm reactors [3,5,6]. Although there is another group of microorganisms called chemoautotrophs that can utilise various gas substrates, there are no reports on their use in these bioreactors.

Chemoautotrophs can oxidise inorganic chemical substances such as hydrogen (H_2_) as their energy source and use carbon dioxide (CO_2_) as the main source of carbon [7]. Although this microorganism offers an opportunity to valorise waste CO_2_, the overall reaction faces challenges such as low solubility of gas in liquid media and usage of explosive gas mixtures. Membranes have the potential to help achieve high gas transfer efficiencies at low gas supply rates due to the high specific surface area available for transfer. In the case of chemoautotrophs, oxygen and the carbon source (CO_2_) can be supplied using membranes which would lead to unique biofilm structures. Possible nutrient profiles for the traditional biofilms and chemoautotrophs are shown schematically in Figure 1. Furthermore, using dense membranes for gas transfer through diffusion can prevent explosive environments that may occur with bubbling. Thus, membrane biofilm reactors are suggested as a promising reactor system for gas fermentation using chemoautotrophic microorganisms.

In this study, biofilm characteristics of a chemoautotrophic bacteria, *Cupriavidus necator* H16, was investigated using a single tube membrane biofilm reactor. The mass transfer coefficient for O_2_ was measured, and a dimensionless model was developed to predict the effect of operational conditions on mass transfer characteristics. The effect of biofilm thickness on substrate diffusion rates and oxygen uptake rate was also investigated using the same membrane setup. This is the first study to investigate biofilm characteristics of a chemoautotrophic bacteria on a tubular membrane and it aims to be the basis of future studies with membranes as gas transfer devices for cultivating *Cupriavidus necator* H16.

## 2. Materials and Methods

### 2.1. Membrane Biofilm Reactor Design

**Figure 2:**
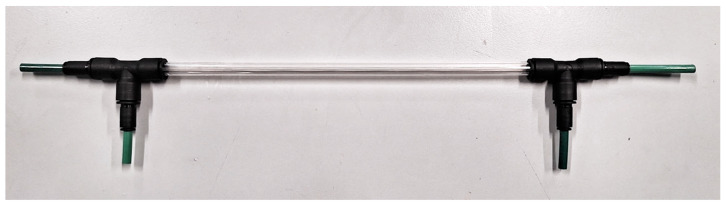
Membrane bioreactor module used in the study.

A tubular non-porous polydimethylsiloxane (PDMS) membrane (Fisher Scientific Ltd., Loughborough, UK) was used in this study to construct the membrane biofilm reactor (see Figure 2). The tubular membrane was potted in a 6 mm nylon tubing using translucent epoxy resin (MG Chemicals, Santa Clara, CA, USA). Membrane biofilm reactor was constructed using a glass column with a 10 mm outer diameter and 8 mm inner diameter with two T-shaped push-in fittings (RS) at the ends. The membrane module with the single PDMS tube was fitted into the glass column by connecting it to the T-shaped push-in fitting with a reducer to fit a 6 mm nylon tubing end. The perpendicular ends of the T-shape push-in fittings were used to connect the liquid inlet and outlet using 6 mm outer diameter Tygon tubing was to minimise possible gas transfer through the tubing. Gas lines were connected to the membrane module using 6 mm nylon tubing. Details of the membrane biofilm reactor are summarised in Table 1.

### 2.2. Oxygen Transfer Rate Measurement

**Figure 3:**
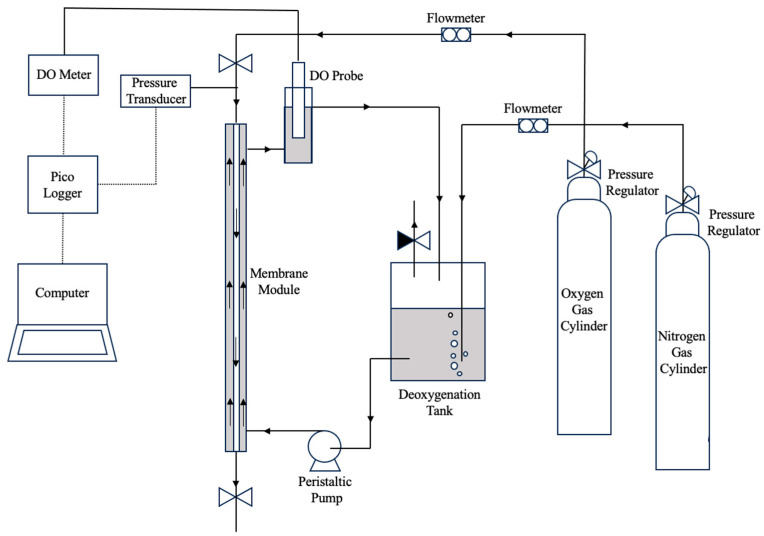
Experimental setup schematic diagram for OTR measurements.

Oxygen transfer rate (OTR) was measured using two methods with an experimental setup shown in Figure 3. In the first method (the dynamic method), a dissolved oxygen (DO) probe (CellOx^®^ 325, WTW, Los Angeles, CA, USA) was used to measure the oxygen concentration in the liquid. For abiotic experiments, deionised water was pumped through the shell side of the membrane bioreactor using a peristaltic pump (Watson Marlow 323, RS, Manchester, UK). The deionised water was deoxygenated by sparging with pure nitrogen until the DO reading reached 0–1 mg/L. Subsequently, pure oxygen was introduced to the lumen of the membrane fibre at a pressure of 90 mbar g and a minimum flowrate of 300 mL/min to maintain the pressure within the fibre during the experiment. The DO probe was fitted into a separate tube and connected to the membrane biofilm reactor outlet to measure the dissolved oxygen concentration over time. Oxygen concentration readings were collected using a Pico Logger and plotted as a function of time until the DO concentration reached saturation point. The rate of oxygen transferred into the liquid was calculated using the following linearised mass balance equation [8]:

(1) $$ln(\frac{C^{*}-{\;C}_{0}}{C^{*}-{\;C}_{1}}{)=k}_{L}a\;\times \;t$$

where C* is the DO saturation concentration at 20 °C, and k_L_a is the volumetric mass transfer coefficient.

The second method for measuring OTR is referred as the pressure drop method [9] whereby two valves are connected at either end of the membrane module. Oxygen gas was introduced to the lumen of the membrane at a minimum flowrate of 200 mL/min, then the valves were closed to maintain the oxygen pressure in the membrane and allow oxygen to leave the lumen only through the membrane. High sensitivity pressure measurements were achieved by using a pressure transducer connected to a Pico Logger to record the pressure drop. For a given intramembrane pressure, the difference between the initial and final pressures indicates the change in mass of oxygen inside the membrane tube [10]. The OTR was calculated by using the pressure drop and converting it to moles by using the ideal gas law. The gas volume was calculated by combining the volume inside the membrane fibre with the tubing between the valves and it remained constant for all experiments. OTR was measured using this method when the biofilm was present on the membrane.

**Figure 4:**
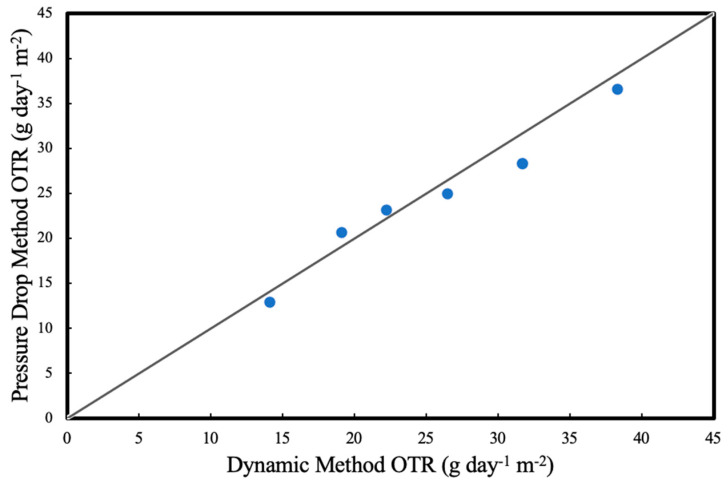
Pressure drop method and the dynamic method oxygen transfer rate comparison at the same operational conditions.

For both methods, each measurement was taken at least in triplicates and measurements were performed in random order. Results for OTRs for deionised water (abiotic experiments) at different liquid recirculation rates were compared in Figure 4. The statistical error in the OTR measurements were found to be less than 5%.

### 2.3. Mass Transfer Model Development

Mass transfer in gas–liquid membrane systems occurs across three parts: the gas layer, membrane layer and liquid layer. The overall mass transfer coefficient can be described based on the general resistance in series model [11]:

(2) $$\frac{1}{K}=\frac{1}{k_{m}H}+\frac{1}{k_{l}}+\frac{1}{k_{g}H^{\mathrm{cc}}}$$

where K is the overall mass transfer coefficient (m/s), k_m_ is the mass transfer coefficient through the membrane (mol m^−2^ Pa^−1^ s^−1^), H is the Henry’s solubility constant (Pa m^3^ mol^−1^), k_l_ is the mass transfer coefficient at the liquid side (m/s), k_g_ is the mass transfer coefficient through the gas side (m/s) and H^cc^ is the dimensionless Henry’s constant. The literature suggests that gas mass transfer resistance is negligible when compared to membrane and liquid resistance [11,12]. The membrane mass transfer coefficient for a nonporous membrane is defined as [13]:

(3) $$k_{m}=\frac{P}{\delta }$$

where P is the permeability of the gas through the membrane (mol m^−1^ Pa^−1^ s^−1^) and δ is the membrane thickness (m).

The mass transfer model developed follows the following assumptions:

1. Membrane temperature and flow conditions are at steady state;
2. Ideal gas law is used for all gas streams;
3. Gas is physically absorbed/desorbed in the liquid; chemical reactions are negligible;
4. Axial concentration gradients in the gas and liquid streams are negligible;
5. No diffusional resistance in the gas side of the membrane.

Using these assumptions, the overall mass transfer coefficient was calculated using the nondimensional Sherwood (Sh) number correlation [14].

(4) Sh=A Rex Scy

A is a constant and Sherwood, Schmidt (Sc) and Reynolds (Re) numbers are calculated as follows [14]:

(5) $$\mathrm{Sh}=\frac{{K\;d}_{\mathrm{eff}}}{D}$$

(6) $$\mathrm{Sc}=\frac{\mu }{D\rho }$$

(7) Re=ρudeffμ

where μ is the viscosity (kg m^−1^ s^−1^), ρ is the fluid density (kg/m^3^), u is the velocity of the fluid (m/s), D is the diffusion coefficient (m^2^/s) and d_eff_ is the module effective diameter calculated as [14]:

(8) $$d_{\mathrm{eff}\;}=\frac{d_{\mathrm{column}}{}^{2}-{\;\mathrm{nd}}_{\mathrm{fiber}}{}^{2}}{d_{\mathrm{column}}{+\mathrm{nd}}_{\mathrm{fiber}}}$$

To calculate an effective Reynolds number, the effective diameter was used when calculating the average velocity of the fluid. Schmidt number is independent of the membrane configuration, and it is the same for all operating conditions. The exponent y in Equation (2) is generally 0.33 in the literature and the same is assumed in this study [14]. For the membrane biofilm reactor constructed, the exponent x and A is calculated using the data for O_2_ via the dynamic method and minimising the error between Equations (4) and (5).

### 2.4. Biofilm Development

A chemoautotrophic microorganism *Cupriavidus necator* H16 was used in this study. Overnight cultures were prepared using 50 mL nutrient-rich LB media in 250 mL Erlenmeyer flasks at 30 °C. For biofilm experiments, minimal salts media (MSM) was used and prepared using (per litre) 9 g Na_3_PO_4_·12H_2_O, 1.5 g KH_2_PO_4_, 1 g NH_4_Cl, 200 mg MgSO_4_·7H_2_O and 1 mL of trace elements that include 4 g ZnSO_4_·7H_2_O, 1 g MnCl·4H_2_O, 0.2 g Na_2_B_4_O_7_·10H_2_O, 0.3 g NiCl_2_·6H_2_O, 1 g Na_2_MoO_4_·2H_2_O, 1 g CuCl·2H_2_O and 7.6 g FeSO_4_·7H_2_O per litre. After autoclaving at 121 °C for 15 min, the media was supplemented with fructose as the carbon source to reach an initial concentration of 15 g/L.

The single-tube membrane biofilm reactor was operated in batch mode for approximately 30 h, until the optical density (OD) value reached 2.5–3.0. The medium was continuously recirculated at a flowrate of 50 mL/min using a peristaltic pump (Watson Marlow 323, RS) and air was fed into the membrane at 300 mL/min. After the initial bacterial attachment on the membrane, the setup was switched to continuous operation mode at very low liquid flowrates of 2 mL/min to avoid cell washing. The reactor was operated for 6 days in continuous mode to grow biofilms. Experiments were repeated four times. The initial biofilm formation was observed after 24 h for each experiment.

### 2.5. Biofilm Thickness Measurement

Biofilm thickness was measured using ImageJ to analyse photos of the biofilm every 24 h for 6 days. Biofilm thickness was measured from multiple sections for each photo that was taken every 24 h, and the average biofilm thickness was calculated for each time point. Since the biofilm obtained is fragile, photos were taken in situ as a non-disruptive technique. Due to the magnifying effect of the glass, each biofilm measurement was corrected by measuring the silicone tube without any biofilm attached using the same method.

### 2.6. Substrate Uptake Measurement

OTR was measured using the pressure drop method every 24 h at different intramembrane pressures and average oxygen uptake rate (OUR) was calculated at different biofilm thicknesses. Residual fructose was determined using a high-performance liquid chromatography (HPLC) system equipped with RID-10A refractive index detector (Shimadzu, Kyoto, Japan) at 50 °C. A total of 20 μL of the sample was injected into the column after the culture supernatant was filtered through Mini-UniPrep syringeless filter devices (Agilent, Santa Clara, CA, USA). The fructose was analysed by Aminex-87H column (Bio-Rad, Watford, UK) at 40 °C. The samples in the column were eluted with 0.014 N H_2_SO_4_ at a flow rate of 0.55 mL/min and pressure of 4.3 MPa. A standard curve for fructose was used for quantification. Using the change in residual fructose concentration, fructose utilisation rate was calculated.

## 3. Results

### 3.1. Effect of Intramembrane Pressure on Oxygen Transfer

**Figure 5:**
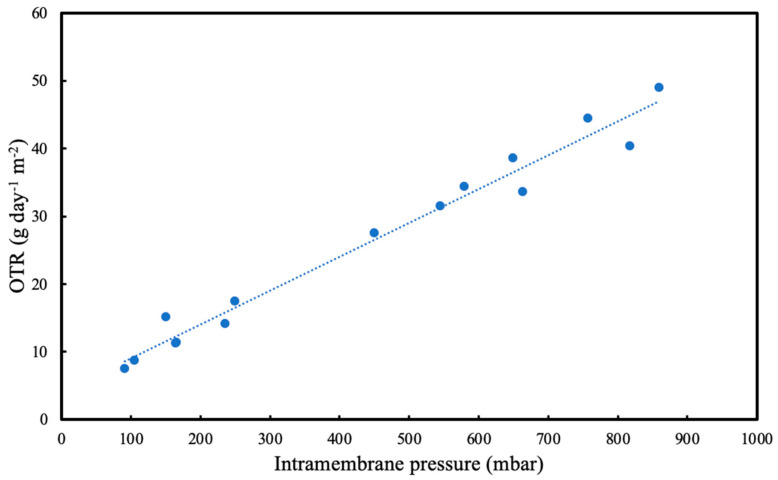
Effect of intramembrane pressure on the oxygen transfer rate calculated using pressure drop method.

Figure 5 shows the effect of intramembrane pressure on OTR using the pressure drop method. In the set of experiments reported here, the OTR is proportional to the intramembrane pressure, and it is higher at elevated intramembrane pressures. As the intramembrane pressure increases, the amount of oxygen at the lumen side of the membrane increases which creates a higher driving force for gas transfer. Thus, the OTR becomes higher at higher intramembrane pressures. Figure 5 further shows that the setup used for OTR calculations using the pressure drop method is robust since the linear correlation is observed with the experimental data.

### 3.2. Mass Transfer Model

The experimental Sherwood number was calculated using Equation (4) and the OTR data obtained above. Since the Sc number is constant, Sh/Sc^0.33^ was plotted against Re number to calculate component x in Equation (4). The constant A was then calculated from the intercept of the graphs. As the component x and A were determined, following correlation was proposed for this system:

(9) Sh=0.38 Re0.36 Sc0.33

**Figure 6:**
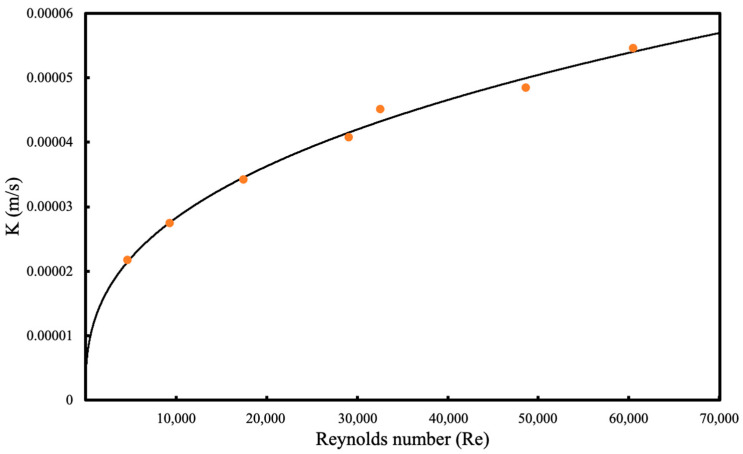
Mass transfer coefficient for O_2_ as a function of the Re. Circles represent the experimental values using the pressure drop method and the solid line represents the predicted model.

Experimental K values for O_2_ data as a function of Re was plotted in Figure 6 with the proposed model.

The maximum mass transfer coefficient was observed at the highest liquid flowrate. As the Re increases, the mass transfer coefficient increases but the slope of the graph begins to decrease. This can be explained by the liquid boundary layer where K becomes independent of the Re at high liquid flowrates. Similarly, as the Re decreases, K tends towards zero which indicates that the liquid boundary layer is the dominant resistance.

### 3.3. Biofilm Development

**Figure 7:**
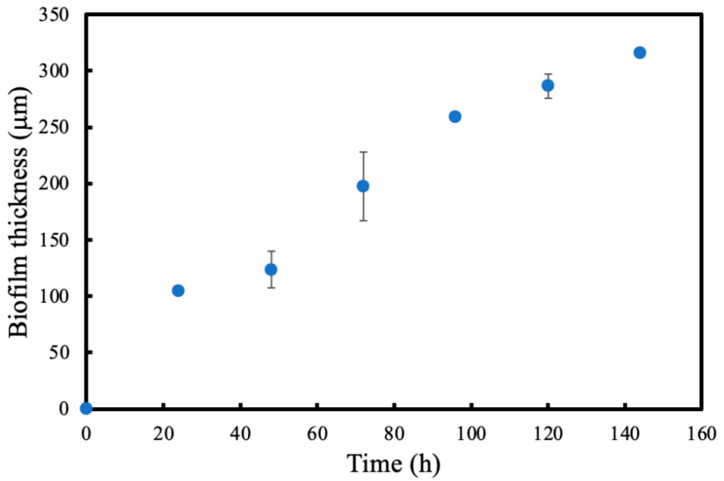
Average biofilm thickness development over time.

**Figure 8:**
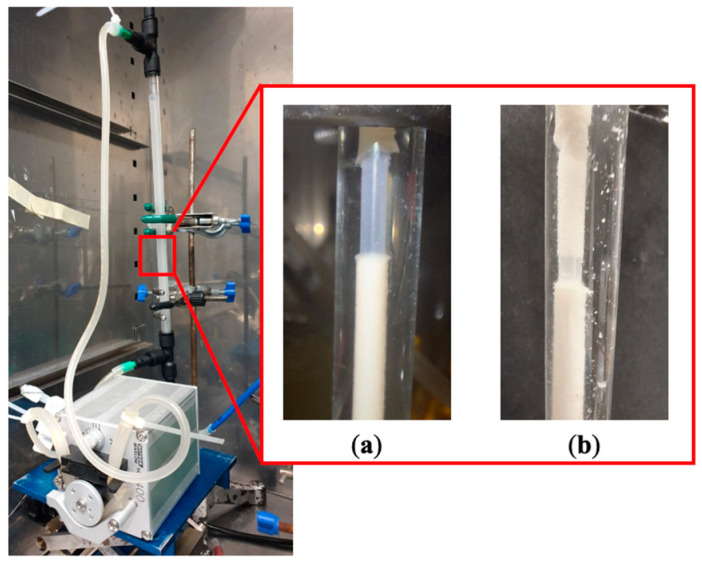
(**a**) Sloughing of biofilm on single tube after 120 h; (**b**) regrowth of biofilm on the sloughed areas in the same region after a further 24 h.

Figure 7 shows the development of average biofilm thickness with respect to time. Sloughing was observed approximately 120 h for each experiment where a small section of biomass along the single tube fibre detached uniformly, shown in Figure 8a. For each experiment, the length of biofilm that sloughed off was between 10 and 20% of the total membrane length. However, regrowth of biofilm in the region of the sloughed areas were rapid. Figure 8b shows the regrowth of biofilm after 24 h. Nevertheless, average thickness measurements at the 144 h point were performed on the parts where no sloughing occurred.

**Figure 9:**
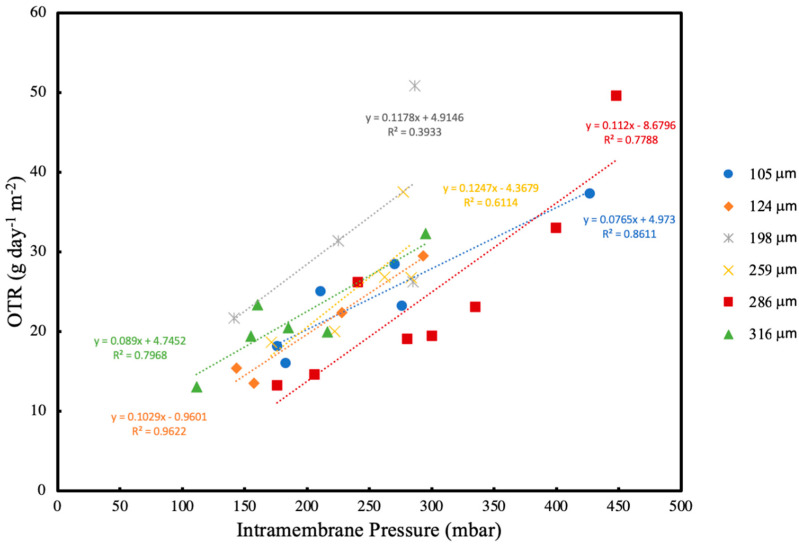
Oxygen transfer rate for biofilm thickness with different intra-membrane pressures.

At 24 h intervals, the OTR was measured at both the normal (steady state) intramembrane pressure (200 mbar) and also at a range of elevated intramembrane pressures (ranging from 120 to 450 mbar). Figure 9 shows the effect of intramembrane pressure on OTR corresponding to a range of different biofilm thicknesses as the experiment progressed. OTR increases linearly as intramembrane pressure increases, which is not unexpected as the driving force for oxygen transfer increases. This suggests that the system is not oxygen limited for the range of oxygen pressures and biofilm thicknesses tested.

**Figure 10:**
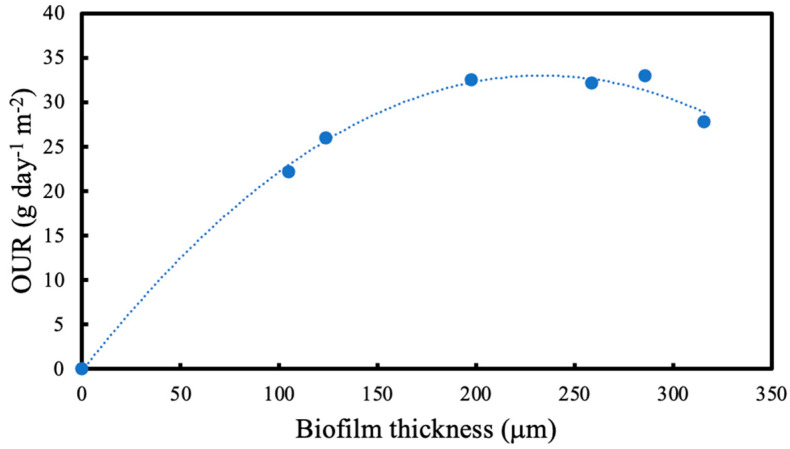
Average oxygen uptake rate measurements at the specified biofilm thicknesses.

Figure 10 shows the average oxygen utilisation rate (OUR) results for measured biofilm thickness. OUR increases as the biofilm thickness increases, levels and then falls. The trend in OUR with respect to biofilm thickness can be explained as follows. In alignment with previously published results for membrane-attached biofilms [2], the trend shown in Figure 10 is a result of the concept of “optimal biofilm thickness”, a unique feature of biofilms where co-diffusion of limiting substrate occurs. Under constant loading conditions, as the biofilm thickness increases, the biofilm changes from a single-substrate limited regime to a dual-substrate-limited regime. Unlike conventional biofilms, the layer of maximum microbial activity within thinner biofilms may not necessarily be located adjacent to the biofilm-liquid interface and as a result, a diffusion barrier exists that reduces the effective concentration of the carbon substrate.

**Figure 11:**
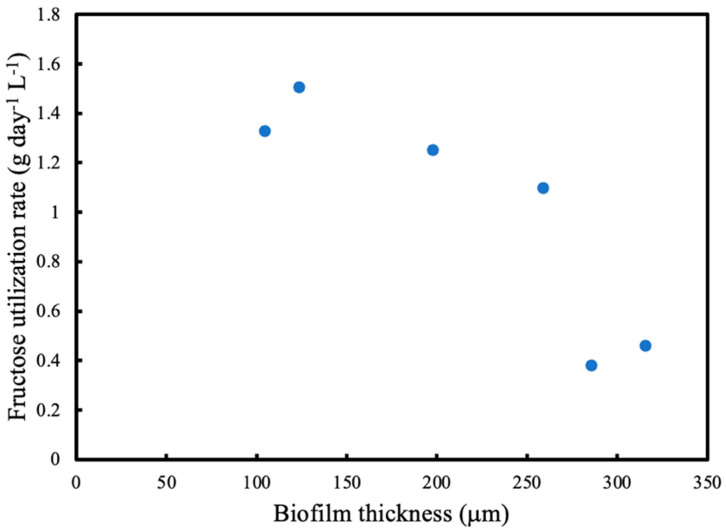
Fructose utilisation rate at corresponding biofilm thickness.

Figure 11 shows the steady state fructose utilisation rate as the experiment progresses with increasing biofilm thickness. The reactor is operated as a batch initially and does not become steady state continuous until 72 h, which corresponds to a biofilm thickness of 200 μm. It can be seen that the fructose utilisation rate starts to decrease between 125 μm and 200 μm and continues to fall significantly as the biofilm thickness progresses. This provides further evidence of the dual-limitation condition that is inherent in membrane attached biofilms.

## 4. Discussion

This study aims to show the initial attachment and biofilm growth of a chemoautotrophic organism *Cupriavidus necator* H16 for proof-of-concept and investigates the gas–mass transfer characteristics of the designed membrane biofilm reactor. PDMS was used as the membrane material for gas transfer due to the high permeability of this material to gas components, especially oxygen [15]. Using dense membranes makes it possible to supply oxygen at high pressures which would overcome the oxygen diffusional limitations in conventional aerated bioreactors and oxygen-transfer efficiencies would approach 100%. Also, dense membranes do not exhibit intrapore fouling and wetting as is the case with microporous membranes [16]. Thus, a PDMS membrane was used in this study for gas transfer.

The Reynolds numbers in this study are in the turbulent range, higher than many studies that investigate the shell side mass transfer correlations. This is due to the small diameter of the membrane bioreactor column used in this study, which increases the liquid velocity within the bioreactor and causes the Reynolds number to be in the turbulent range. Using high Re numbers makes the liquid boundary layer resistance lower than the resistance caused by the membrane layer. Therefore, at elevated liquid velocities, the mass transfer coefficient (K) becomes independent of the Re. In the literature, the power of the Re number was reported between 0.3 and 0.93, which shows that the suggested correlation is comparable with the previously reported studies [14]. Furthermore, the proposed model showed the best fit to the experimental data of the study.

Single tube (or single fibre) membrane systems are useful when designing membrane biofilm reactors. Using a single fibre makes it easier to analyse the performance of the biofilm formed on the membrane. Depending on the results, the system can be scaled up by simply adding more fibres or having multiple single fibre membrane systems in parallel. Furthermore, imaging the biofilm in a single tube system is easier compared to using multiple membranes in a bundle. Biofilm characteristics of the chemoautotrophic bacteria used in this study were investigated by supplying pure oxygen through the single fibre membrane. In the case of *C. necator*, carbon and oxygen can be delivered from the membrane side since this microorganism can utilise CO_2_ as its carbon source. However, the membrane was used to supply only pure oxygen and fructose was used as the carbon source which was present in the liquid media. Thus, the substrate concentration profiles of the biofilm formed should resemble Figure 1b where the highest oxygen concentration is at the biofilm–membrane interface (substratum) and the highest fructose concentration is at the biofilm–water interface.

As the biofilm continues to grow, the dissolved oxygen concentration eventually falls to zero within the biofilm. Since the microorganism used in this study is aerobic, this could potentially lead to oxygen limitation. However, OTR results at various intramembrane pressures and biofilm thicknesses indicate that oxygen limitation is not a concern within the range of testing. Thus, it can be concluded that the resulting biofilm is entirely aerobic, and there were no instances of oxygen limitation throughout the experiments.

Formation of the biofilm on the membrane surface creates an extra mass transfer resistance for oxygen. However, Casey et al. reported that for biofilm thicknesses up to 600 μm, the OTRs are higher compared to abiotic measurements [2]. In this study, when the OTRs are compared in the presence and absence of a biofilm, it is seen that the OTRs are higher until the biofilm thickness reaches 124 μm after 24 h. Since microorganisms are utilising the oxygen provided, the concentration of oxygen within the biofilm decreases. Due to the concentration difference between the biofilm and the membrane lumen, the oxygen flux through the membrane increased.

Sloughing is a common occurrences at later stages of biofilm development and it can be caused by a combination of shear stress and biofilm growth rate [17]. As the biofilm grows, shear stress (or velocity) in the glass column increases as the diameter decreases. In larger bioreactors, the effect of biofilm thickness on velocity can be insignificant since larger vessels are used. However, in the biofilm reactor used in this study, the diameter of the column is relatively small. Thus, biofilm growth can significantly change the shear stress on the biofilm which might have played a role in the sloughing. The maximum thickness reached before sloughing in the present study was approximately 300 μm, which is considered a relatively thin biofilm [18].

In membrane-attached biofilms, biofilm thickness is a major influence on determining the process performance. There is an optimum biofilm thickness for each specific application and condition. An excessively thin biofilm might not provide enough activity where an excessive biofilm proliferation might hinder the reaction by increasing the resistance to diffusion by substrate transfer. For nitrogen removal, Terada et al. showed that the performance of a membrane-aerated biofilm reactor (MABR) is optimal when the biofilm thickness stabilises at approximately 1600 μm [19]. Another study by Matsumoto et al. showed that the nitrogen removal efficiency of an MABR can exceed 70% when the biofilm thickness is between 600 and 1200 μm [20]. Sanchez-Huerta et al. also concluded that biofilm thickness over 580 μm enhances the removal of organic micropollutants in an MABR [21]. These studies show that the biofilm thickness has an important effect on the performance of the membrane biofilm reactors. Therefore, it is important to identify biofilm growth characteristics including biofilm growth and stripping.

In this study, product formation was not considered since the main objective of the study was to investigate the biofilm characteristics and its effect on substrate diffusion rates. The model organism used in this study can produce a polymer that could be used to create bioplastics. However, it is possible to use a genetically modified version of this strain to produce a wide range of products.

This study provides a basis for future studies with biofilm characteristics of *Cupriavidus necator* H16 on gas transferring membranes. Here, the substrates are introduced to the biofilm from opposing sides and the effect of biofilm thickness on oxygen and fructose utilisation rates are discussed. Due to the chemoautotrophic metabolism of this microorganism, more complex bioreaction scenarios can be studied in the future, such as supplying CO_2_ as the carbon source through the membrane.

## 5. Conclusions

The goal of this study is to assess the mass transfer coefficients for O_2_ in a single tube membrane biofilm reactor and investigate the biofilm characteristics of *Cupriavidus necator* H16. Membranes provide better mass transfer rates for low soluble gaseous substrates for gas fermentation. Also, they provide a safe delivery system when explosive gas mixtures are used. The dimensionless model used for predicting O_2_ mass transfer coefficients was validated through experimental measurements. The highest liquid flowrate yielded the highest mass transfer coefficient. After a biofilm of 200 μm biofilm thickness had developed, a decline in both the oxygen uptake rate and fructose utilisation rates was observed, indicating that dual-substrate limitation came into effect after three days of biofilm growth. A decline in biofilm growth rate was observed after 96 h, which corresponds to 260 μm biofilm thickness.

## Figures and Tables

**Table 1:** Details of the membrane biofilm reactor design.

Membrane Material	PDMS
Inner diameter of the membrane fibre (mm)	1.0
Outer diameter of membrane fibre (mm)	2.0
Wall thickness of membrane fibre (mm)	0.5
Effective fibre length (cm)	33
Surface area of membrane fibre (m^2^)	2.07 × 10^−3^
Glass column effective length (cm)	30
Inner diameter of glass column (cm)	0.8

## Data Availability

The data presented in this study are available in the article entitled “Investigating Mass Transfer and Reaction Engineering Characteristics in a Membrane Biofilm Using Cupriavidus necator H16”.
